# Review of tools and algorithms for network motif discovery in biological networks

**DOI:** 10.1049/iet-syb.2020.0004

**Published:** 2020-08-01

**Authors:** Sabyasachi Patra, Anjali Mohapatra

**Affiliations:** ^1^ Department of Computer Science IIIT Bhubaneswar Odisha India

**Keywords:** computational complexity, graph theory, biology, search problems, network size, motif size, network motif discovery, biological networks, network composition, recurrent patterns, over‐represented patterns, local properties, search space, subgraph isomorphism check, NP‐complete problem, NP‐complete problem, exact census, design principles, background algorithms, runtime efficiency, space requirement

## Abstract

Network motifs are recurrent and over‐represented patterns having biological relevance. This is one of the important local properties of biological networks. Network motif discovery finds important applications in many areas such as functional analysis of biological components, the validity of network composition, classification of networks, disease discovery, identification of unique subunits etc. The discovery of network motifs is a computationally challenging task due to the large size of real networks, and the exponential increase of search space with respect to network size and motif size. This problem also includes the subgraph isomorphism check, which is Nondeterministic Polynomial (NP)‐complete. Several tools and algorithms have been designed in the last few years to address this problem with encouraging results. These tools and algorithms can be classified into various categories based on exact census, mapping, pattern growth, and so on. In this study, critical aspects of network motif discovery, design principles of background algorithms, and their functionality have been reviewed with their strengths and limitations. The performances of state‐of‐art algorithms are discussed in terms of runtime efficiency, scalability, and space requirement. The future scope, research direction, and challenges of the existing algorithms are presented at the end of the study.

## 1 Introduction

Technological enhancement in recent years leads to the development of many real‐world complex networks such as biological networks, social networks, power distribution networks, ecological networks (food web), software engineering diagrams, molecular structures, computer networks, electronic circuits, and world wide web (WWW). These networks are built to observe the associated nature and functional behaviour of the constituent elements. In this study, several tools and algorithms for discovering network motifs are reviewed. Network motif is one of the important local properties of biological networks such as metabolic networks, gene regulatory networks, and protein–protein interaction (PPI) networks. These networks provide insight for biological systems to understand the biological functions better. However, the huge size of the biological networks demands efficient computational methods to extract information about how these interactions perform various vital functions.

A graphical representation can depict considerable information about any real‐world network. In the context of network analysis, a graph data structure can be used to model the biological networks. In biology, transcriptional regulatory networks and metabolic networks are usually modelled as directed graphs. For instance, in a transcriptional regulatory network, nodes represent genes, and edges represent the transcriptional relationships between them. In this network, if gene A regulates gene B, then the directed edge starts at A and terminates at B. Understanding interactions between proteins in a cell may benefit from a model of a PPI network. PPI networks are typically modelled as undirected graphs, in which nodes represent proteins and edges represent interactions between the proteins in an organism. There is no direction associated with the interactions in such networks. Metabolic networks describe the biochemical interactions within a cell through which substrates are transformed into products through reactions catalysed by enzymes. Metabolic networks generally require more complex representations, such as hyper‐graphs or bipartite graphs, as reactions in metabolic networks generally convert multiple inputs into multiple outputs with the help of other components. These networks can represent the complete set of metabolic and physical processes that determine the physiological and biochemical properties of a cell. Metabolic networks are complex. There are many kinds of nodes (proteins, particles, and molecules) and many connections (interactions) in such networks.

The properties of biological networks can be classified into two broad categories: global properties and local properties. Global properties are mainly used for network modelling and characterisation. Some of these properties are small‐world property, power‐law degree distributions, clustering coefficients etc. Recently researchers have shifted their attention from global properties to local properties that describe a large complex network as a composition of several small subgraphs. Many real‐world networks contain recurrent patterns that are overrepresented with respect to their appearances in the random networks. These recurrent patterns are described as network motifs by Milo *et al.* [1]. These are recognised as basic building blocks of complex networks and associated with unique functional properties [2]. The study of these network motifs enhances the knowledge of network functions. Network motif is found in several real‐world networks such as PPI [3, 4], gene regulatory network [5–8], social network, WWWeb [9], food webs [10], brain neural network [11], electronic circuits [12], and software life cycle [13].

Network motifs are not only statistically significant but also significant to their respective systems. Higher frequencies of network motifs suggest that these are present due to evolutionary factors and important functionality [1]. Each network has different motifs that are more frequent and thus more important to the system or organism. The characteristics of network motifs are almost similar in all real‐world networks. For example, transcriptional regulatory networks and neuronal connectivity networks have common network motifs known as feed‐forward loops [14] and bifans [14]. This observation suggests that these two networks are similar in some design aspects. However, networks having altogether different characteristics may possess the same network motifs. For example, a feed‐forward loop is thought to be used in information processing [1] and found in neuronal connectivity and gene regulatory networks. However, food web networks that do not deal with information processing possess this network motif. This observation indicates how network motifs are biologically significant in their ability to help analyse, explain, and classify networks. Owing to the significance of network motifs, many efforts have been put forth in developing tools that can discover network motifs.

All network motif discovery algorithms include the following steps:

- All possible subgraphs of a given size are extracted from the input network.
- Frequencies of these enumerated subgraphs are calculated from the input network.
- The statistical significance of each candidate network motifs is determined by comparing their frequencies to those of random networks having the same degree distribution as the input network.

These steps involved a huge computational cost. First of all, the time complexity of enumerating all possible subgraphs in the input network is exponential as the number of alternative patterns increases exponentially along with the increase of network size and motif size. This problem also includes the subgraph isomorphism check, which is NP‐complete [15]. Generating a large number of randomised networks to measure statistical significance multiplies the computational cost many folds. In addition to this, the gradual increase in the size of real networks amplifies the challenges mentioned above. To address these challenges, several algorithms based on heuristics and approximations have been proposed in the literature. The key strategy used by many algorithms is subgraph sampling. Another important strategy is to apply the symmetry breaking policy to reduce isomorphism‐related computations [16]. The pattern growth approach is extensively used to reduce the number of graph isomorphism check. A list of existing literature reviews is presented in Table 1.

**Table 1:** List of reviews on network motif discovery

Year	Authors	Tools and algorithms reviewed	Contributions and limitations
2008	Ciriello and Guerra [17]	MFinder, Enumerating Subgraph (ESU), Rand‐ESU, and NeMoFinder	this survey present a summary of motif discovery algorithms for PPI networks only. It is necessary to study other biological networks which have different topological characteristics.
2009	Ribeiro *et al*. [18]	MFinder, FAst Network MOtif Detection (FANMOD), and Grochow	this survey limited to three methods only, and the performance of these methods are relatively low compared to the state‐of‐art algorithms
2010	Wong and Baur [19]	MFinder, FANMOD, Grochow–Kellis, MODA, NeMoFinder, Kavosh, and MAVisto	this is a survey on various tools and algorithms for network motif discovery, which include the experimental data and limitations of these algorithms. Experimental data from various tools are provided in this study, including runtimes for different subgraph sizes, network sizes, number of random networks generated, different frequency measures.
2011	Wong *et al*. [20]	MFinder, FANMOD, Grochow–Kellis, MODA, NeMoFinder, Kavosh, and MAVisto	this is a survey on motif detection, specifically in the biological network. This review briefly explained the various methods without including the corresponding algorithmic details.
2012	Masoudi‐Nejad *et al*. [21]	MFinder, ESU (FANMOD), Grochow–Kellis, MODA, NeMoFinder, Kavosh, FPF (MAVisto), and G‐tries	this is a survey on computational aspects of major network motif discovery algorithms with their merits and limitations. However, the algorithm details are missing in this review.
2014	Tran *et al*. [22]	MFinder, FANMOD, Grochow–Kellis, MODA, NeMoFinder, Kavosh, MAVisto, NetMODE, Acc‐Motif, and QuateXelero	this survey presents a study on 11 essential tools and algorithms for network motif discovery. This survey compares these tools and algorithms and investigates their key features. Classification of network motifs, biological significance, and the applications of network motifs are discussed. This survey also includes future research directions for network motif discovery. However, this study does not explain the algorithms with suitable examples, and experimental data from various tools are not provided.
2015	Kavurucu [23]	MFinder, FANMOD, Grochow–Kellis, MODA, NeMoFinder, Kavosh, and MAVisto	this is a review on various network motif discovery algorithms with an appropriate example network. However, this study does not perform runtime analysis of the existing algorithms.
2016	Salari *et al*. [24]	MFinder, FANMOD, Grochow–Kellis, MODA, NeMoFinder, Kavosh, MAVisto, G‐tries, and QuateXelero	this survey demonstrates the importance of network motif discovery and discusses the techniques available to solve this problem. The available algorithms are classified using a simple framework, and their strengths and weaknesses are explored considering the experimental and laboratory data. This review also skips the algorithm details.

In this study, network motif discovery tools and algorithms are extensively reviewed, and comprehensive analysis has been done. Various strategies are discussed with their strengths and limitations. The algorithms are explained with pseudo code and classified based on their strategy. This review also includes the experimental results derived from a few comparative studies in the literature. Finally, the challenges involved in network motif discovery and future research directions in this area are discussed.

The rest of the paper is organised as follows: Section 2 briefly discusses some of the applications of network motifs in various fields. Section 3 introduces the various concepts related to network motif discovery. The strategies used by various tools and algorithms are discussed in Section 4. Section 5 classifies the algorithms into various categories. Section 6 summarises the performance, strengths, and weaknesses of major motif discovery algorithms. Section 7 presents the dataset and experimental results derived from a few comparative studies in the literature. Conclusion and future research directions are discussed in Section 8.

## 2 Some applications of network motifs

Network motifs find important applications in network modelling, performance analysis of biological networks, network homology detection, protein function annotation, superfamily classification, complex prediction, disease discovery, drug design, network resource management etc. Some of these applications are summarised here.

- *Network modelling:* Network motifs provide a better understanding of the modularity as well as the large‐scale structure of the complex biological network [25]. The artificial network model can be created from the real‐world networks utilising the network motifs.
- *Performance analysis*: The primary information storage units in biological and artificial networks are directed feedback loop and feed‐forward loop. Hence, identification of these motifs helps to understand why some recurrent neural networks are known for excellent memory performance [26].
- *Network homology detection*: Network motifs are used for network homology detection [1].
- *Function annotation*: The labelled network motifs found in the PPI networks can be used to predict the functions of the unknown proteins. In this application, network motifs are discovered based on the structure and biological meanings. The identified network motifs are labelled so that they can be used to predict the functions of unknown proteins in the PPI networks [27]. The functional roles of some genes in gene regulatory networks may be better understood from network motifs. For instance, the identification of microRNA motifs in gene regulation networks improves the understanding of their functional roles [28].
- *Superfamily classification*: Network motifs are used for superfamily classification [29], as similar network possesses similar network motifs. Pržulj *et al.* [30] used network motifs as features to classify PPI networks. These features are also used to validate the PPI in the PPI networks [3].
- *Complex prediction*: Features extracted from network motifs can be used to predict protein complexes in PPI networks.
- *Disease discovery:* Network motifs are used for cancer disease diagnosis [31], prediction of survival possibility in breast cancer [32], drug repositioning [33], functional behaviour in diabetes patients [34] etc. The three‐node network motifs found in the human signalling network have been screened for identifying breast cancer samples from normal samples [35]. The accuracy of this method is good enough for breast cancer diagnosis and therapy, as well as other types of cancer [35]. The identification of the network motifs explores the mechanisms of cervical carcinoma response to epidermal growth factor in regulation networks [36].
- *Network resource management*: Network motifs are mapped to applications for identifying network activity in network resource management and security enforcement [37].
- *Protocol identification*: Application protocols in network traffic are identified using network motifs. This application supports network administrators to secure and manage network resources [38].
- *Validity of evolutionary trees*: The evolutionary trees are built based on the character overlap graphs. In this application, the correctness of evolutionary trees is validated by finding under‐represented network motifs called holes in the character overlap graphs [39].
- *Clustering of proteins:* Features extracted from network motifs can be used for clustering the proteins in an interaction network [40].

## 3 Basic concepts for network motif discovery

Network motif discovery is the process of finding subgraphs whose frequency is statistically significant within a complex network. The primary tasks involved in this process are frequency computation, random graph generation, statistical significance measure, subgraph isomorphism etc. The concepts related to these tasks are discussed below.

### 3.1 Graph

A graph G=(V,E) comprises a set of vertices V={v} and a set of edges E⊆(V×V). An edge e=(u,v)∈E connects a pair of vertices *u* and *v*. The vertices *u* and *v* are adjacent to each other. Networks are represented as graphs.

### 3.2 Induced graph

**Fig. 1:**
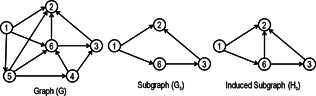
Graph G with non‐induced subgraph G_s_ and induced subgraph H_s_

A subgraph $G_{s}=(V_{s},E_{s})$ of G=(V,E) consists of a subset of nodes $V_{s}\subseteq V$ and a subset of edges $E_{s}\subseteq E\cap (V_{s}\times V_{s})$ connecting the nodes of $V_{s}$ in the original graph. This subgraph $G_{s}$ is said to be induced when it contains all the edges (u,v)∈E for all $u,v\in V_{s}$. Fig. 1 presents an example of a graph along with induced and non‐induced subgraphs.

### 3.3 Subgraph isomorphism

**Fig. 2:**
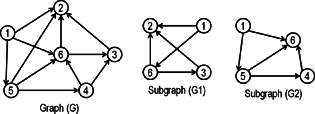
*Isomorphic subgraphs G1 and G2 with bijective function* f:G1→G2 *defined as* 1→1, 2→6, 6→5 *and* 3→4

Two graphs G=(V,E) and $H=(V^{\prime },E^{\prime })$ are said to be isomorphic if there exists a bijective function *f* between *V* and $V^{\prime }$ such that for each edge (u,v)∈E there exist an edge $(f(u),f(v))\in E^{\prime }$. Checking graph isomorphism is an NP‐hard problem. However, finding a subgraph of *G*, which is isomorphic to *H*, is an NP‐complete problem. The computational cost of this problem can be reduced by using heuristic approaches such as the canonical labelling of a graph. McKay [15] developed a well‐known method for isomorphism testing known as NAUTY. This method represents a graph in the form of a canonical label. Two isomorphic graphs must have the same canonical label [41]. A hypothetical graph and its two isomorphic subgraphs are shown in Fig. 2.

### 3.4 Subgraph frequency

**Fig. 3:**
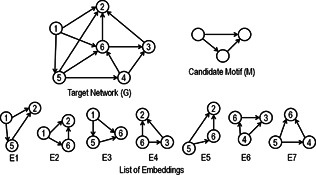
*E1 to E7 are list of embeddings of a candidate motif (M) in a target network G. For frequency concept* F1, *the set* E1−E7 *represents all matches, so* F1=7. *For* F2, *one of the three possible sets* {E1,E4,E7} *or* {E1,E5,E6} *or* {E3,E4,E7} *can be taken, so* F2=3. *Finally, for the frequency concept* F3, *only one set* {E1,E6} *is allowed, therefore* F3=2

The subgraph frequency of a network motif represents the number of embeddings of that subgraph present in the target network [42]. The frequency measure depends on how the nodes and edges are allowed to overlap among the embeddings of subgraphs [43, 44]. Based on the overlapping of graph elements, three frequency concepts are derived, such as F1, F2, and F3. The F1 measure allows the overlapping of both vertices and edges among the instances of the subgraph, whereas only vertices can be shared in F2 measure. Hence the subgraph instances of F2 measure are edge‐disjoint. The frequency measure F3 computes disjoint subgraph instances in which no sharing of vertices or edges are allowed. Downward closure property is satisfied by both F2 and F3 measures. These frequency concepts are illustrated in Fig. 3 and Table 2. The selection of a specific frequency concept directly affects the statistical significance measures such as *z*‐score and *P*‐value [17]. Sometimes it is required to find overlapping embeddings and, sometimes, only non‐overlapping network motifs are significant. Therefore, frequency concepts play a vital role in the design of motif discovery algorithms [14, 19].

**Table 2:** Listing of embeddings (as shown in Fig. 3) based on frequency concepts F1, F2, and F3

Frequency concept	Sharing of vertices	Sharing of edges	Frequency	Selected embeddings
F1	yes	yes	7	{E1, E2, E3, E4, E5, E6, E7}
F2	yes	no	3	{E1, E4, E7} or {E1, E5, E6} or {E3, E4, E7}
F3	no	no	2	{E1, E6}

### 3.5 Random networks

Random networks are used to measure the statistical significance of network motifs. Barabasi and Oltvai [45] provide an introduction to network models and their properties. Some of the relevant random graph models are discussed here. Random networks preserve the degree distribution of biological networks [46]. The degree distribution P(k) of a network represents the probability of a node having a degree *k*. Most of the biological networks have power‐law degree distribution [47] that follow a power‐law $P(k)∼k^{-\gamma }$, where γ is the power‐law exponent. These networks are also known as scale‐free networks. Random networks are generated by using common randomisation techniques such as switching method, stubs method, and the ‘go with the winners’ method [48].

*Switching method*: The switching method implements the Markov chain technique. It uses the nodes of the input network, preserves their in‐degree and out‐degree, and switches the edges between the nodes numerous times to obtain randomisation. This method randomly selects two edges, u→v and x→y, in the network, and exchanges the ends to form two new edges u→y and x→v. This process preserves the in‐degree and out‐degrees of the nodes. The limitation of the switching method is that the time required for proper mixing is not known for the Markov chain [49]. However, it has been experimentally verified that a network with an *E* number of edges achieves adequate randomisation by 100×E times of switching [49].
*Stubs method:* The stubs method keeps the same in‐degrees and out‐degrees of the nodes of the input network. Each node has in‐stubs and out‐stubs for in‐degrees and out‐degrees of the node, respectively. A matching algorithm is used for the pairing of in‐stubs and out‐stub. Theoretically, this creates random edges between nodes while still preserving the in and out degrees of all nodes. The method discards any self‐edges or multiple edges. This becomes a problem because numerous real‐world networks have multiple edges between two nodes [49].
*Go with the winners' method:* The ‘go with the winners’ algorithm starts with multiple graphs. It then carries out the stubs method. The algorithm periodically copies all of its graphs to compensate for the eliminated graphs due to self‐edge or multiple edges. This maintains the average number of graphs to remain constant. The process stops after linking all the stubs. This algorithm can be very slow, especially with large‐scale networks [49].

**Fig. 4:**
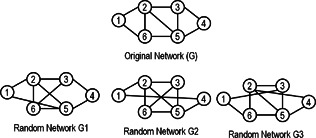
Illustration of random networks preserving degree distribution of the original network

The switching algorithm is probably the algorithm of choice for random graph generation that samples correctly in the limit of long times and practice is found to give good results when compared with other [46]. Some of the random networks preserving degree distribution of the original network are shown in Fig. 4.

### 3.6 Statistical significance

Network motif discovery not only finds the frequent subgraph but also validate their uniqueness using the uniqueness threshold. A subgraph is frequent if its appearance in the graph is above a threshold value. On the other hand, a subgraph is said to be unique, if its frequency is statistically significant. A few measures related to this are given below.

#### 3.6.1 Frequency threshold

The frequency threshold requires that the frequency of a subgraph in an input network ($f_{\text{input}}$) must exceed a threshold frequency (*F*). On the other hand, the uniqueness threshold requires that the frequency of a candidate motif must be a certain level higher than its mean frequency in a set of random networks. Let a size‐*k* subgraph $g_{k}$ occur $f_{t}$ times in the input network. Let $f_{r}$ be the mean of frequencies of $g_{k}$ in the random networks and *U* be the uniqueness threshold. Then, $g_{k}$ is said to be unique if it satisfies the following condition;

(1) $$f_{t}-f_{r}>U\times f_{r}$$

#### 3.6.2 Significance metrics

A list of significance metrics are given below:

*z‐score*: A candidate motif is said to be statistically overrepresented if its *z*‐score is above 2.0 [41]. *z*‐score of a network motif with frequency $f_{t}$ in the target network, mean frequency $f_{r}$ and standard deviation $\sigma_{r}$ in a set of random networks can be defined as
(2) $$z-\text{score}=\frac{f_{t}-f_{r}}{\sigma_{r}}$$
*P‐value*: A network motif is said to be statistically significant if the *P*‐value of its frequency is <0.01. If *n* represents the number of times the frequency of the candidate motif in random networks exceeds its frequency in the target network out of *N* number of random networks, then the *P*‐value of the network motif can be defined as
(3) $$P-\text{value}=\frac{n}{N}$$
*Significance profile*: The significance profile of a set of network motifs is a vector of *z*‐scores [29]. The significance profile of the ith network motif ($SP_{i}$) with *z*‐score $z_{i}$ in a set of *n* number of motifs can be calculated as
(4) $$SP_{i}=\frac{z_{i}}{\sqrt{\sum_{j=1}^{n}z_{j}^{2}}}$$
*Concentration:* The concentration of a candidate motif in a network denotes how frequent it is in comparison with other subgraphs of the same size [50, 51]. Specifically, if there are *n* number of size‐*k* subgraphs in a network, then the concentration ($C(g_{k,i})$) of the ith subgraph $g_{k,i}$ is defined as
(5) $$C(g_{k,i})=\frac{f_{k,i}}{\sum_{j=1}^{n}f_{k,j}}$$
  where $f_{k,i}$ denotes the frequency of the subgraph $g_{k,i}$.
*Abundance:* The abundance (Δ) of a network motif is a metric similar to the *z*‐score [29] and is defined as
(6) $$\Delta (g_{k})=\frac{f_{t}-f_{r}}{f_{t}+f_{r}+\epsilon }$$

For small frequencies, ϵ prevents the abundance approaching infinity.

## 4 Network motif discovery process and strategies

An induced size‐*k* subgraph $\left\{G_{k}\right\}$ of graph *G* is called a network motif for a given set of parameters {*P*, *U*, *D*, *N*} [18] if it satisfies the following conditions:

- Over‐representation: $\text{Prob}({\bar{f}}_{r}(G_{k})>f_{t}(G_{k}))\leq P$
- Minimum frequency: $f_{t}(G_{k})\geq U$
- Minimum deviation: $f_{t}(G_{k})-{\bar{f}}_{r}(G_{k})>D\times {\bar{f}}_{r}(G_{k})$

Milo *et al.* [1] used {0.01, 4, 0.1, 1000} as the set of parameters {*P*, *U*, *D*, *N*}, but depending on the requirements, other combinations may be used.

The network motif discovery process is used to find all *k*‐node subgraphs {*G_k_*} with 3≤k≤K occurring in *G* such that the frequency or concentration of $G_{k}$ is above the given frequency threshold *F* and significantly higher than that in the random networks. The confidence level *P* represented in terms of either *z*‐score or abundance or Significance Profile (SP) [20], here *G* is input network represented as a directed or undirected graph; *K* is the maximum size of the network motif to be searched; *P* is the confidence level; *F* is the frequency threshold; *U* is the uniqueness threshold; and *N* is the number of random networks.

**Fig. 5:**
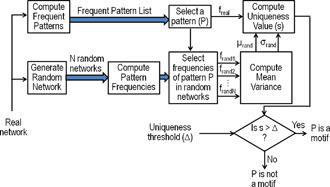
Block diagram of network motif discovery process

Fig. 5 presents a generic block diagram to discover network motifs in an input network.

Various strategies used in the network motif discovery process are discussed here. For example, the pattern growth approach is used to generate all possible subgraphs. Enumeration or sampling or mapping is used for subgraphs census. Canonical labelling or symmetry breaking coupled with mapping is used for isomorphism checking. Table 3 illustrates these strategies with reference to various tools and algorithms.

**Table 3:** Summary of strategies adopted by different tools and algorithms

Tools/algorithms	Year of publication	Strategy used	Subgraph census	Frequency concept	Network type	Isomorphism checking
MFinder [48, 50]	2005	pattern growth	exact census (enumeration) and edge sampling	F1	undirected and directed	NAUTY
MAVisto [44, 52]	2005	FPF	exact census (enumeration)	F1, F2, F3	undirected and directed	NAUTY (canonical labelling)
FANMOD [51, 53]	2005	pattern growth	exact census (enumeration) and node sampling	F1	undirected and directed	NAUTY (canonical labelling)
NeMoFinder [54]	2006	tree filtering search, graph cousins	exact census (enumeration)	F1	undirected	NAUTY (canonical labelling)
Grochow and Kellis [16]	2007	Automorphisms (NAUTY)	exact census(mapping)	F1	undirected and Directed	mapping with symmetry‐breaking
Kavosh [41]	2009	pattern growth	exact census(enumeration)	F1	undirected and directed	NAUTY (canonical labelling)
MODA [42]	2009	ET	exact census(mapping), sampling	F1	undirected	mapping with symmetry‐breaking
G‐tries [55]	2010	G‐tries	exact census(enumeration, mapping)	F1	undirected	NAUTY (canonical labelling)
NetMODE [56]	2012	pattern growth	exact census(enumeration)	F1	undirected and directed	stores all canonical labels in memory
Acc‐Motif [57]	2012	combinatorial acceleration	exact census(enumeration)	F1, F2	undirected and directed	no isomorphism check
QuateXelero [58]	2013	quaternary tree	exact census(enumeration)	F1	undirected and directed	NAUTY (canonical labelling)
Elhesha–Kahveci [59]	2016	pattern join	exact census(enumeration)	F2	undirected	NAUTY (canonical labelling)
ParaMODA [60]	2017	ET	exact census(mapping)	F1	undirected	mapping without symmetry‐breaking
NemoMap [61]	2018	ET	exact census(mapping)	F1	undirected	mapping with symmetry‐breaking
MOtif Discovery using Expansion Tree (MODET) [62]	2018	ET	exact census(mapping)	F1	undirected	mapping with symmetry‐breaking
Motif discovery using Dynamic Expansion Tree (MDET) [63]	2019	DET	exact census(mapping)	F2	undirected	mapping with symmetry‐breaking
pattern‐join [64]	2019	pattern join	exact census(enumeration)	F2	undirected and directed	NAUTY (canonical labelling)

### 4.1 Pattern growth strategy

Pattern growth strategy can be used to systematically generate all possible size‐*k* subgraphs starting with a base graph [44]. The base graph is extended one step at a time by adding a node or an edge and uses the extended subgraph to generate further variants. A tree data structure is used for systematically performing these tasks. Every node of the tree represents a subgraph. The subgraph represented by a parent node is obtained by the extension of the subgraph represented by the child node. A pattern growth tree built for size‐*k* subgraphs can be used to systematically enumerate all appearances of size‐*k* subgraphs present in the input network. Restrictions are imposed in the extension process of the pattern growth tree to confirm that each subgraph appears only once. Flexible pattern finder (FPF) motif analysis and visualisation tool (MAVisto) uses a pattern tree to generate higher‐order patterns from the generating parent of less number of edges [52]. Kavosh built an implicit tree with restrictions to ensure that each subgraph is enumerated only once, which leads to an improvement in both time and memory [41]. MODA uses this strategy and reduces the computational cost significantly [42]. Downward closure property can be used for frequency measure F2 to prune branches that are rooted in a node whose subgraph frequency fails to reach the threshold. The MODET and MDET algorithms also use this strategy.

### 4.2 Subgraph census: exhaustive search

Subgraph census is the process of enumerating all occurrences of subgraphs by scanning the input network node‐by‐node or edge‐by‐edge. Milo *et al.* [1] in 2002 discovered the network motifs by performing an exhaustive recursive search. This method can find all connected induced and non‐induced subgraphs. Another exact search algorithm is ESU [65] that generates size‐*k* subgraphs starting with a node and adds nodes one by one incrementally to reach the required size. The algorithm maintains a list of candidate nodes for future additions to the partially generated subgraph. This list is dynamically updated by adding the nodes whose label is higher than the nodes present in a partially constructed subgraph, and they are adjacent to the nodes already in the subgraph. Full enumeration algorithms are extremely time‐consuming. They require a large number of computations due to the exponential increase of different isomorphic subgraphs with respect to motif size and network size. It is also necessary to find the frequencies of each different isomorphic subgraph in both the target network and the randomised networks [19].

### 4.3 Subgraph census: sampling

Kashtan *et al.* [50] adopt a probabilistic approach to extract subgraph samples from the input network and compute the subgraph frequencies by taking an adequate number of random samples. The sampling method is faster than full enumeration and insensitive to the size of the input network [19], which make it enable to discover larger motifs. It has been observed that closely accurate results can be obtained by taking the right amount of trials. However, there is a chance of missing some potential motifs with non‐zero probability.

#### 4.3.1 Edge‐sampling

An edge‐sampling strategy [50] randomly picks an edge from the target network and iteratively expands that to a size‐*k* subgraph by randomly selecting new edges adjacent to the nodes already in the sample. When the size of the sample subgraph reaches *k*, all the edges connecting its nodes in the input network are added to get an induced subgraph. The MFinder tool uses this strategy. This edge‐sampling strategy is biased [18] as the probability of sampling different size‐*k* subgraphs is not uniform even if they have the same topology. Also, there is a chance of recounting the same subgraphs multiple times. This strategy assigns a probabilistic weight [18, 19] to each subgraph to overcome this limitation. However, it leads to excessive memory usage. This strategy can extract rare motifs with high probability and relatively very less number of samples required for this process (even with 5000 samples from a transcriptional network of *Escherichia coli* (423,519), MFinder find the concentration of motifs similar to the exact census). However, this strategy does not scale well with large (size‐8) motifs [54].

#### 4.3.2 Node‐sampling

The drawback of the edge‐sampling strategy is overcome in the node‐sampling strategy [51, 53]. The node‐sampling is able to pick the size‐*k* subgraph with a uniform probability. This strategy probabilistically traverses the pattern growth tree that guarantees that the size‐*k* subgraph present at the leaf nodes will be explored with equal probability. To avoid redundant computation, it ensures that a particular subgraph will be encountered exactly once. The FANMOD and MODA use the node‐sampling strategy as it is significantly efficient than edge‐sampling. Although this strategy is fast, it has limitations such as the empirical calculation of probabilities. It avoids picking of nodes that do not belong to a connected component and hence do not identify a subgraph.

### 4.4 Subgraph census: mapping

The mapping strategy [16] takes a size‐*k* query graph and maps it onto the input network to find all instances of that graph. This strategy is in contrast to enumerating the input network to find all size‐*k* subgraphs and then classifies them into embeddings of non‐isomorphic size‐*k* candidate motifs. A mapping strategy allots a rank to the nodes in the input network based on their degree properties and then map them to the nodes in the candidate motif [16] having similar characteristics. Grochow and Kellis [16] proposed this strategy, and MODA [42] also adopt this strategy. The unique feature of this strategy is to resolve the isomorphism check issue partially by applying the mapping with symmetry breaking [16]. The major limitation of this strategy is to generate all variants of query graphs when its size exceeds 10, as the computations become intractable [66].

### 4.5 Symmetry breaking

**Fig. 6:**
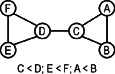
*Symmetry breaking conditions that will break all the symmetries of a six‐node graph, courtesy of Grochow and Kellis* [16 *]*

The symmetrical structure of a graph can be seen as self‐isomorphisms, which is also known as automorphisms. The set of subgraphs satisfying the automorphisms requirement belong to the same equivalence class. Each equivalence class can be represented as a set of symmetry breaking conditions [16]. These symmetry breaking conditions reduce the number of isomorphism checks significantly. Grochow and Kellis proposed this strategy, which is often used with a mapping strategy. Symmetry breaking conditions for a six node symmetric graph is shown in Fig. 6.

### 4.6 Flexible pattern finder (FPF)

**Fig. 7:**
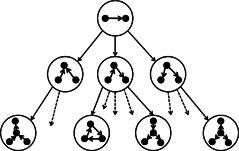
*Example of a pattern tree (size‐3 patterns are partially shown), courtesy of Schreiber and Schwobbermeyer* [44 *]*

This strategy searches for a pattern in a pattern growth tree with maximum frequency under a given frequency concept [52]. The node of the pattern growth tree represents the patterns that are supported by the input network. The root node is the simplest possible pattern with two vertices connected with an edge. The child node is obtained by adding an edge with the parent pattern. For isomorphism checking, a canonical label is assigned as an identity of each pattern, and it removes duplicity. The algorithm does not extend a branch further if the frequency of the corresponding pattern falls below the frequency of a pattern of target size. If there is a nearly maximum frequent pattern of target size discovered early in the search tree, then it discards the intermediate size patterns before saturation. These criteria reduce the search space significantly [44]. However, motif discovery not only required the pattern with maximum frequency but also required patterns with a frequency higher than the threshold value. Pruning criteria can be applied for frequency concepts F2 and F3 to reduce the search space by cutting the sub‐trees rooted at a node whose frequency is below a threshold. However, it cannot be applied for the F1 measure. Fig. 7 illustrates the concept of a pattern tree for directed subgraphs.

### 4.7 Tree filtering search

This strategy is applicable for undirected networks only [54]. In this process, repeated size‐*k* trees are extracted, then they are used to partition the input network. Subsequently, the graph join operation is performed to compute the frequency of size‐*k* subgraphs. NemoFinder [54] uses this strategy. The input network is naturally partitioned into a set of subgraphs by the repeated trees. Hence, the problem of counting the subgraph frequency is reduced to counting the number of subgraphs in the above set, which is naturally downward closed. The graph cousins are used to facilitate the candidate generation process and graph join operation. However, generating cousins is ambiguous, and it may find several redundant isomorphic subgraphs.

### 4.8 Expansion tree (ET)

**Fig. 8:**
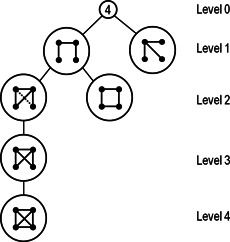
*ET of size‐4 subgraph, courtesy of Omidi et al.* [42 *]. At the first level, there are non‐isomorphic size‐4 trees, and at each level, an edge is added to the parent subgraph to form a child graph. All subgraphs are non‐isomorphic to prevent redundancy*

The concept of ET was proposed by Omidi *et al.* [42]. The ET plays a vital role by providing query graphs systematically from minimally connected size‐*k* trees to a complete graph. In this strategy, at first, the frequency of size‐*k* trees is computed in the target network and then expands these trees edge by edge until a complete graph such that there is no room for new edges. Hence the frequency of other query graphs can be computed by mapping them to the nodes of ET without using subgraph isomorphism. A size‐4 ET is shown in Fig. 8.

### 4.9 G‐tries

**Fig. 9:**
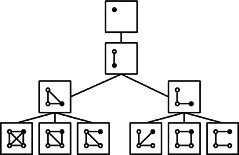
*Tree representing a set of six graphs. Each tree node adds a new vertex (in black) to the already existing ones in the ancestor nodes (white vertices), courtesy of Ribeiro and Silva* [55 *]*

A g‐trie is a multiway tree that can store a set of graphs [55]. Each node of the g‐trie contains information about a single vertex of a subgraph and its corresponding edges to the vertices of its ancestor g‐trie node. A path from the root node to the leaf node corresponds to a single graph. Descendants of a g‐trie node share a common subgraph. A size‐4 g‐trie is shown in Fig. 9. Backtracking is used to estimates the frequency of induced subgraphs in a target network. This strategy takes advantage of the common substructure of several different candidate subgraphs (all the descendants) that leads to partial isomorphic match during the search process.

### 4.10 Combinatorial acceleration

Acc‐Motif [57] uses this strategy to improve computational complexity. This strategy assigns an integer variable to each isomorphic pattern and increments it directly. It is not required to perform any isomorphism check using NAUTY. Only size‐3 and size‐4 network motifs are extracted using this strategy.

### 4.11 Quaternary tree

A quaternary tree represents the data structure of a rooted tree [58]. Each internal node of this tree can have a maximum of four children. A node can have at most five neighbours; one among them is the parent node, and the rest four are children. The edges of the tree are labelled with a number or character, or any other symbol. A labelled quaternary tree can be searched using a given string that consists of the same set of symbols used for labelling that tree. The search process is initiated at the root node. In each step, a symbol is read from the input string, and the current pointer moves to the child node of the corresponding symbol. If a child does not exist corresponding to the symbol, then a new node is created for that symbol, and the current pointer moves to that child node. The search process terminates when the input string is read completely. This strategy eliminates the number of NAUTY calls significantly by using the tree data structure and hence improves the performance. The QuateXelero algorithm [58] uses this strategy.

### 4.12 Pattern joining

This strategy is based on basic building patterns, and the graph joins operations [59]. The basic building patterns act as a guide to construct larger patterns through iterative pattern join operation. The subgraphs of the current set of patterns are joined with the subgraphs of the basic building patterns to construct larger patterns iteratively. At the end of the iteration, the resulting set of patterns becomes the current set of patterns for the next‐join iteration. Two subgraphs need to share at least one edge to participate in the join operation. The joining of two such subgraphs either yields a pattern that is isomorphic to one of the existing patterns or a new one. Elhesha and Kahveci [59] and Patra and Mohapatra [64] use this strategy.

## 5 Classification of network motif discovery tools and algorithms

**Fig. 10:**
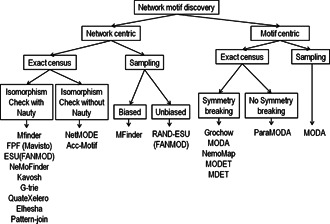
Classification tree of network motif discovery algorithms

Network motif discovery tools and algorithms are broadly classified into two categories; network‐centric and motif‐centric. These algorithms are further classified into various subcategories depending on the adopted strategy, such as sampling or exhaustive census. A broad level classification tree is shown in Fig. 10.

### 5.1 Network‐centric tools and algorithms

Network‐centric tools and algorithms enumerate the entire network and do the subgraph census to compute the frequency of the subgraphs. The subgraphs that do not occur in the target network are never encountered in this type of algorithm. Network‐centric algorithms can be further classified into exact census or sampling. The exhaustive enumeration algorithms are extremely time‐consuming and costly as this process required for both the target network and the randomised networks. Also, the number of isomorphic subgraph types increases exponentially with the increase of motif size. Algorithms that use the exact census are MAVisto, NeMoFinder, Kavosh, G‐trie, QuateXelero, Elhesha–Kahveci, and Pattern‐join.

Kashtan *et al.* [50] developed a probabilistic algorithm to estimate subgraph frequencies by sampling subgraphs. The sampling approach is fast as compared to full enumeration. The runtime does not increase asymptotically with the increase of subgraph size and network size. Hence the sampling method can identify larger network motifs than exhaustive enumeration methods. A major problem with Kashtan *et al.* method is that it has bias sampling [42] as the subgraphs are not sampled with uniform probability [18]. This problem is addressed by assigning a weight to each subgraph with a value of 1/(sampling probability of the subgraph) [18]. Algorithms that use the sampling approach are MFinder and FANMOD. These algorithms also have the option to perform exhaustive census in the input network.

### 5.2 Motif‐centric tools and algorithms

Motif‐centric tools and algorithms allow for a single specific query graph to be examined [18] for a potential network motif. Grochow–Kellis and MODA use this approach. These algorithms compute the frequency of size‐*k* query graphs directly by mapping them to the input network. The NAUTY tool is used to generate all possible size‐*k* subgraphs. Motif‐centric algorithms can be further classified into exact census or sampling depending on the strategy used for counting subgraph frequencies [20]. The sampling strategy is used by MODA to speed up the overall network motif discovery process. The efficiency of these algorithms is further improved by mapping with symmetry breaking techniques. Other motif centric algorithms are network motif mapping (NemoMap), ParaMODA, MODET, and MDET. All of these algorithms use symmetry breaking techniques except ParaMODA.

## 6 Review of network motif discovery tools and algorithms

The first major contribution to network motif discovery is proposed by Milo *et al.* [1]. Schreiber and Schwobbermeyer [44] propose different frequency concepts for computing pattern frequency in a FPF algorithm. Wernicke [65] proposed a specialised algorithm ESU that could avoid redundancy in computation through proper enumeration of network motifs. NeMoFinder is proposed by Chen *et al.* [54] for finding mesoscale network motifs. Grochow and Kellis [16] proposed the first motif centric algorithm, where frequency counting is done on a specific isomorphic class. Kashani *et al.* [41] brought a new network‐centric algorithm named as Kavosh to improve runtime efficiency. Omidi *et al.* is the second motif centric algorithm proposed MODA [42], which is based on a pattern growth methodology. G‐trie [55] is a specialised data structure developed in 2010 for finding network motifs in undirected graphs. Network motif detection (NetMODE) [56] is a network motif detection software package developed in 2012 to improve runtime efficiency. Accelerated motif (Acc‐Motif) [57] is the first network motif discovery algorithm based on a combinatorial approach. QuateXelero [58] is an efficient network motif detection algorithm developed in 2013. A novel algorithm proposed by Liang *et al.* [67] named as CoMoFinder to accurately and efficiently identify composite network motifs in genome‐scale co‐regulatory networks. Nikam and Chauhan [68] designed a new algorithm using a suffix‐graph data structure to retrieve the subgraph efficiently that detects network motifs. Elhesha and Kahveci [59] proposed a motif centric algorithm (Elhesha–Kahveci) for finding disjoint network motifs in a target network. ParaMODA [60] and NemoMap [61] improve upon the motif‐centric tool Grochow–Kellis and MODA [60]. Lin *et al.* [69] present a novel study on network motif discovery using graphical processing units (GPUs). Chen and Chen [70] published an efficient sampling algorithm for network motif detection. Lin *et al.* [69] in 2017 used GPUs to study network motifs. Hu and Shang [71] in 2017 proposed a novel graph canonisation algorithm for detecting network motifs from transcriptional regulation networks. Luo *et al.* [72] in 2018 proposed an efficient network motif discovery approach for co‐regulatory networks. Patra and Mohapatra [62] proposed an efficient and scalable motif centric algorithm (MODET) based on a static ET. The space limitation of MODET is removed by MDET [63] by using a dynamic ET (DET). Fast and scalable network motif discovery is proposed by Wang *et al.* [73] in 2009 for exploring higher‐order network organisations. Patra and Mohapatra [64] propose a network‐centric algorithm in 2019 using the pattern join method. Briefing of some of the major network motif discovery tools and algorithms are listed below.

**Fig. 11:**
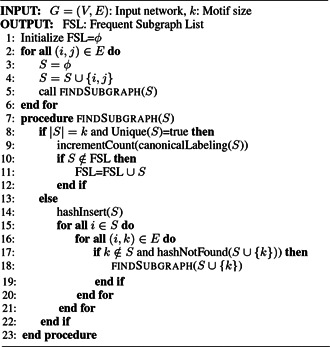
Algorithm 1: MFinder enumeration algorithm

*MFinder:* MFinder is the first motif discovery tool that implements both the exact census and the sampling technique for enumerating the subgraphs in the input network. The exact census method is proposed by Milo *et al.* [1] in 2002, which is a recursive backtracking search. Algorithm 1 (see Fig. 11) describes this method with pseudocode. MFinder required a lot of space to maintain the associated hash tables, and hence it is incapable of finding large motifs.

**Fig. 12:**
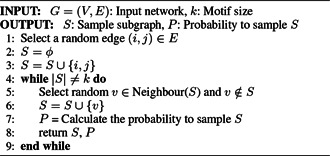
Algorithm 2: MFinder sampling algorithm

Kashtan *et al.* [50] proposed a probabilistic approach for sampling the subgraphs instead of exhaustive enumeration. They have performed edge‐sampling in the input network to find the concentrations of induced subgraphs. The pseudocode of the MFinder sampling algorithm is presented in Algorithm 2 (see Fig. 12). Sampling makes this algorithm enable us to deal with large networks and able to discover large network motifs that cannot be enumerated using the exact census. MFinder can accurately estimate the subgraph concentration in a network that has a very low concentration. The execution time of the sampling method is independent of network size. However, the sampling method is biased because all the subgraphs may not have an equal probability of being sampled [50]. This algorithm has tried to overcome this problem by assigning a weight of W=1/P to the sampled subgraph, where *P* represents the sampling probability of the subgraph. This method can successfully discover network motifs up to size‐6 using exact census and up to size‐8 using sampling.

*Limitation:* MFinder uses the biased sampling strategy developed by Wang and Bour [19]. All the enumerated subgraphs are stored in memory, and the same subgraphs may be considered repeatedly. The sampling technique does not consume less time than the exact census for a network without hubs. The sampling approach may not discover the complete set of network motifs. As the sampling approach is biased, the same subgraphs may appear multiple times that cause redundancy. MFinder is unable to deal with large subgraphs due to the exponential sampling procedure [50].

*FPF (MAVisto):* MAVisto [52] is based on a FPF algorithm proposed by Schreiber and Schwobbermeyer [44]. This algorithm exploits the downward closure property, which is applicable for frequency concepts F2 and F3. The downward closure property ensures that the frequency of subgraphs decreases monotonically with respect to the increase of subgraph size. FPF is based on a pattern tree that can hold different isomorphic patterns. The structure of a pattern tree is explained in Section 4.6. The FPF algorithm does not consider the infrequent subgraphs, and the enumeration process terminated quickly by avoiding unnecessary computation. FPF is most useful for frequency concepts F2 and F3 because pruning the branches of pattern tree using the downward closure property of these frequencies [74] reduces the search space significantly. Lookup table for isomorphic checking [52] makes this algorithm fast for detecting motif of sizes 3–5. This method can successfully discover network motifs up to size 7.

*Limitation:* MAVisto is inefficient in counting subgraph frequency due to its complex approach and can only discover small motifs of size up to 7. MAVisto is even slower than MFinder because MFinder uses a sampling technique, whereas MAVisto enumerates all possible subgraphs in a search space that increases exponentially with respect to network size. Downward closure property is not applicable to the F1 frequency concept.

**Fig. 13:**
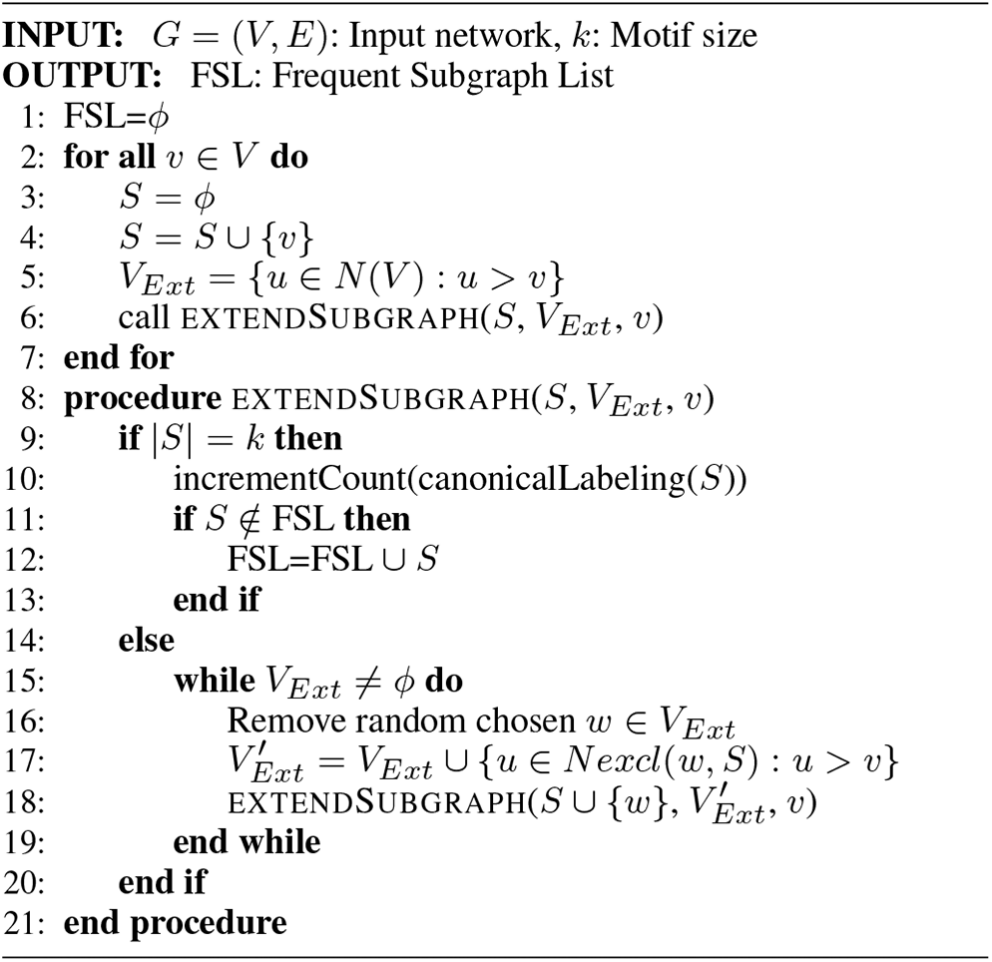
Algorithm 3: ESU enumeration algorithm

*ESU (FANMOD)*: FANMOD toll is implemented based on the ESU algorithm. The ESU is an exact census algorithm which can avoid symmetries and search all subgraphs only once [51, 53]. Algorithm 3 (see Fig. 13) describes ESU with pseudocode. The canonical graph labelling algorithm NAUTY [15] is used by FANMOD for subgraph classification [51]. FANMOD determines the significance of subgraphs using an analytical approach called DIRECT. Hence, it does not require the classical null‐model of random networks.

**Fig. 14:**
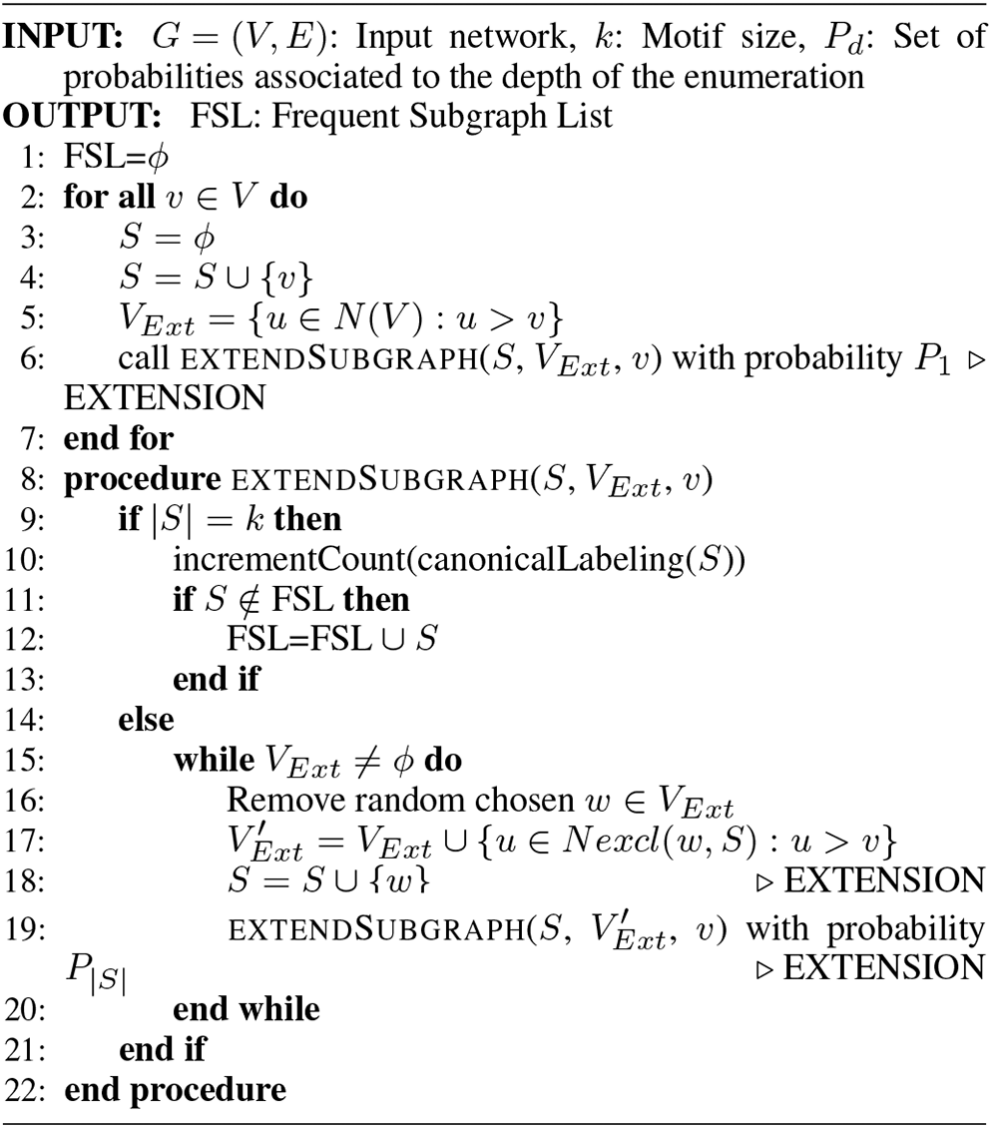
Algorithm 4: FANMOD sampling algorithm

Wernicke [53] proposed an algorithm Random (RAND)‐ESU that provides a significant improvement over ESU. This is implemented in the FANMOD [51] tool. RAND‐ESU effectively exploits an unbiased node sampling throughout the network and assures counting subgraphs only once. FANMOD uses an unbiased node sampling strategy instead of edge sampling, which makes sure each subgraph counted only once. Similar to ESU, this algorithm also starts with a root node and maintains a list of possible neighbours for the extension. The FANMOD sampling algorithm chooses each size‐*k* subgraph with a certain probability [53], and it assures that all the subgraphs are sampled with equal probability. Unlike the Kashtan *et al.* algorithm, FANMOD is unbiased [18, 19]. The RAND‐ESU algorithm is described in Algorithm 4 (see Fig. 14). This tool provides an option to take either an exact census or a uniform sample. FANMOD is much more efficient than exhaustive search algorithms due to counting of every subgraph just once and can identify motifs up to size eight [51] using sampling. This method can successfully discover network motifs up to size 8 using the exact census.

*Limitation:* The memory usage of FANMOD increases remarkably with the increase of subgraph size and network size [41]. Hence, it can discover motifs up to size 8. For highly concentrated subgraphs, the null‐model of random networks is faster than the DIRECT method. In addition to this, it is unable to extract non‐induced subgraphs.

**Fig. 15:**
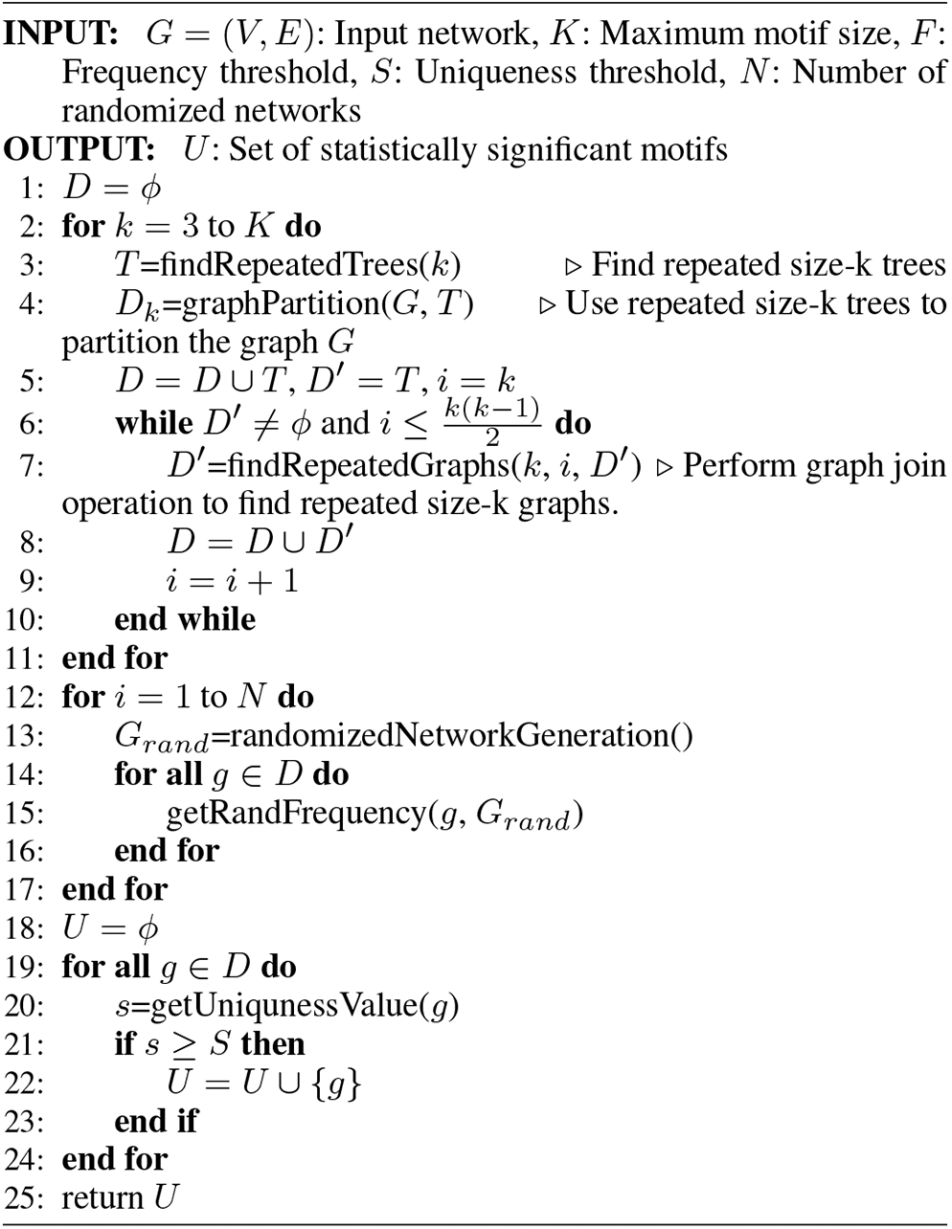
Algorithm 5: NeMoFinder algorithm

*NeMoFinder*: NeMoFinder was proposed by Chen *et al.* [54] based on the idea presented in SPanning tree based maximal graph mINing (SPIN) [75], which first extracts frequent trees then expands them into non‐isomorphic graphs [17]. The frequent size‐*k* trees are used to divide the input network into a set of size‐*k* graphs. Frequent size‐*k* subgraphs are generated by joining a subgraph with its derivative subgraphs, also known as cousin subgraphs. The new graph generated is an edge advanced in comparison with its parent subgraph. The pseudocode of NeMoFinder is shown in Algorithm 5 (see Fig. 15). NeMoFinder searches the repeated trees using the same technique as SPIN [75]; then, these trees are extended to subgraphs at a very low‐cost [54]. NeMoFinder can detect motifs up to size 13 with 20–100‐fold speed up as compared to the predecessor.

*Limitation*: NeMoFinder can detect motifs only in undirected PPI networks. The generation of graph cousins is not simple, and it is derived from a canonical representation of a graph which is not closed under join operation. There is no precise method to derive cousins from a graph, and joining a subgraph with its cousins leads to redundancy in generating a particular subgraph more than once.

**Fig. 16:**
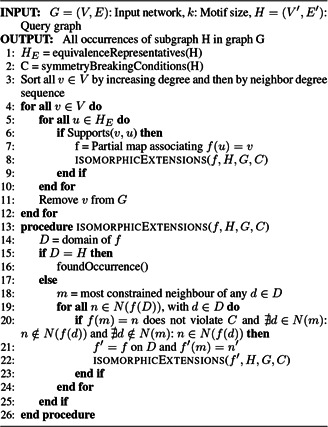
Algorithm 6: Grochow enumeration algorithm

*Grochow and Kellis*: Grochow and Kellis [16] proposed the first motif‐centric algorithm for exhaustively enumerating subgraphs in an input network. The frequency of a query graph is exhaustively determined by mapping it into the input network. The algorithm first computes a set of symmetry‐breaking conditions for each query graph. Then the branch‐and‐bound technique is applied to find all possible mappings from the query graph to the input network, which satisfies the required symmetry breaking conditions. The mapping always begins from one representative of each equivalence class. The symmetry‐breaking conditions are computed by finding automorphisms of a given graph using McKay's tools [15, 76]. The symmetry‐breaking conditions eliminate the requirement of isomorphism check and hence reduce the additional need of time and memory. Algorithm 6 (see Fig. 16) presents the pseudocode for this algorithm. The algorithm has an exponential speedup by pruning the search space using the symmetry‐breaking technique that eliminates repeated isomorphism check. In addition to that, the subgraph hashing technique significantly improves the performance of subgraph isomorphism check [16]. Grochow–Kellis can find motifs up to size 7 using the exact census and can map an undirected query graph of size up to 31. The number of subgraphs encountered without symmetry‐breaking is up to 100 times more than with symmetry‐breaking. Grochow–Kellis achieve an exponential improvement in runtime over the two versions of Milo *et al.* algorithm.

*Limitation*: Grochow–Kellis may process some subgraphs as candidate motifs that might not be present in the input network. For large motif size, it is impossible to generate all possible isomorphic classes of query graphs.

*Kavosh:* Kavosh algorithm consists of four major parts such as (i) enumeration, (ii) classification, (iii) random graph generation, and (iv) motif identification [19, 41]. A tree data structure is used to enumerate the presence of all size‐*k* subgraphs in the input network. Kavosh is based on pattern trees with added constraints. The algorithm starts with a node that can be expanded to a size‐*k* subgraph, then that node is removed from the input network, and the process repeated for remaining nodes [41]. To eliminate redundant computations, each specific tree is constructed only once [19]. Other constraints confirm that the numerical level of all the children of a specific tree must be higher than the label of the root node of the tree. NAUTY is used for isomorphism checking during subgraph enumeration. Kavosh can detect motifs up to size 12, and it is faster than MAVisto and MFinder. For specific networks, it is also quicker than FANMOD.

*Limitation*: Kavosh becomes slower with the increase of motif size due to the exponential growth of subgraph isomorphism testing for subgraph classification.

**Fig. 17:**
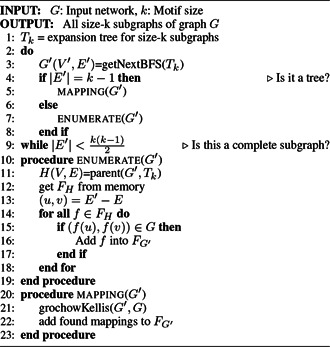
Algorithm 7: MODA enumeration algorithm

*MODA*: MODA [42] uses the concept of the ET to compute the frequency of query graphs. The algorithm starts by computing the frequencies of the size‐*k* subtree in the input network, and then these subtrees are expanded by adding edges according to the growth of the ET $T_{k}$. The structure of the ET is discussed in detail in Section 4.8. For each graph size, a separate ET needs to be built. The ET uses a static data structure that can be stored and retrieved as required [19, 42]. Similar to Grochow and Kellis algorithm, MODA also utilises symmetry‐breaking conditions. MODA calls the Grochow and Kellis algorithm in its first level of the ET to compute the frequencies of size‐*k* subtrees. To compute the frequencies of subgraphs present at the higher levels, MODA uses the mapping strategy and exploit the information content of parent subgraphs. The pseudocode of MODA is detailed in Algorithm 7 (see Fig. 17). The mapping module store the computed mapping in the memory and use this information to compute the frequency of non‐tree query graphs by taking *O*(1) steps. The pseudocode of MODA is detailed in Algorithm 7 (Fig. 17). MODA also adopts the sampling strategy to reduce the computational cost with the expense of accuracy [19, 42]. This method can successfully discover network motifs up to size 9 using the exact census and up to size 10 using sampling. MODA outperforms existing algorithms and able to extract both induced and non‐induced subgraphs.

*Limitation*: MODA can discover network motifs only in undirected networks. All possible size‐*k* subgraphs need to be stored in the ET. Hence the algorithm runs out of space to save the ET for motif size beyond 10. Another drawback of this algorithm is the requirement of huge memory to store the embeddings of the parent subgraph.

*G‐tries*: In 2010, Ribeiro and Silva proposed a novel data structure for storing a collection of sub‐graphs, called a g‐trie [55]. G‐trie data structure is a prefix tree, i.e. built based on common substructures of subgraphs and support partial isomorphic match for several different candidate subgraphs at a given time [21]. This data structure stores the subgraphs according to their structures and finds their occurrences in the input network. For the network motif discovery, this data structure is built only for the subgraphs present in the input network. The constructed tree is reused for the computation of subgraph frequencies in the random network, which significantly improves the computational efficiency. The detailed construction of a g‐trie is explained in Section 4.9. After constructing a g‐trie, the counting takes place. A backtracking technique is used to count subgraph frequencies, which is similar to the technique employed by other motif‐centric approaches such as MODA and Grochow and Kellis algorithms. This algorithm takes advantage of common substructures, which ensures a partial isomorphic match for several different candidates subgraphs at a given time [21]. The sharing of common substructures in a g‐trie reduces the memory requirement of the prefix tree significantly. It also uses the symmetry‐breaking strategy to eliminate the over counting of subgraphs. The random networks are searched only for the subgraphs that are present in the input network. G‐trie is able to identify network motifs up to size 9 faster than FANMOD [51] and Grochow–Kellis [16].

*Limitation*: The prefix tree requires a vast storage space when the motif size and network size increases. This algorithm also wastes a substantial amount of time for searching the subgraphs that end up not existing in the network. This algorithm is completely infeasible for large motif size and applicable only for undirected networks.

*NetMODE*: NetMODE [56] is the first method that performs subgraph isomorphism check without NAUTY [77]. NetMODE has a pre‐treatment phase to store size‐*k* (k≤5) subgraph in memory that avoids the use of NAUTY. The reconstruction conjecture for directed graphs is used for motif size 6. To extend the motif size further (k≥7), in its preprocessing phase, it stores only the canonical labels of the subgraphs that are likely to be encountered in the future. The rest are saved in an auxiliary file. NetMODE minimises the time taken for canonical labelling and performs up to about 30 times faster than its predecessors when k≤5 and up to about 20 times faster when k=6.

*Limitation*: To avoid NAUTY, the algorithm has to use a considerable amount of memory in its preprocessing phase. It is not scalable and can only detect network motifs up to size 6.

*Acc‐Motif*: Combinatorial techniques are used in Acc‐Motif for accelerating the motif discovery process [57]. Acc‐Motif uses independent algorithms for counting isomorphic subgraphs of sizes 3, 4, and 5. Acc‐Motif achieves significant speedup over FANMOD for motif sizes 3 and 4.

*Limitation*: Acc‐Motif is incapable of dealing with motif size beyond 5 using combinatorial techniques.

*QuateXelero*: In 2013, Khakabimamaghani *et al.* proposed a fast network motif detection technique called QuateXelero. QuateXelero uses a quaternary tree data structure to performs partial classification of enumerated subgraphs before calling NAUTY. This algorithm reduces the number of calls to NAUTY for isomorphism check to improve the performance [58]. The QuateXelero algorithm is derived from the ESU (FANMOD) motif detection algorithm and uses a quaternary tree data structure. The functionality of the quaternary tree is explained in Section 4.11. This quaternary tree is used to classify the enumerated subgraphs. This method can successfully discover network motifs up to size 12 using the exact census. The computational time of QuateXelero is much less than Kavosh for all networks. However, it uses a massive amount of memory as compared to Kavosh. QuateXelero is much faster than g‐tries for subgraph census on the original network, but the census in random networks is slow for large motifs.

*Limitation*: QuateXelero consumes a large amount of memory to construct the quaternary tree for detecting large motifs in the input network.

**Fig. 18:**
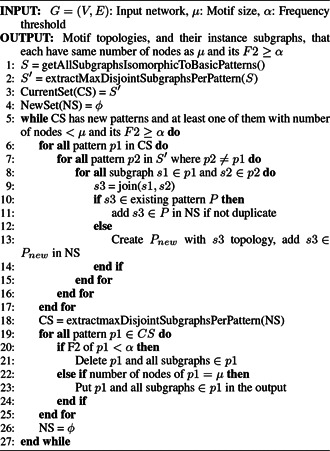
Algorithm 8: Elhesha–Kahveci algorithm

*Elhesha and Kahveci*: Elhesha and Kahveci [59] developed a scalable algorithm to discover large disjoint network motifs. This method uses basic building patterns to generate large patterns through iterative join operation. Any patterns with four or more edges can be generated by joining the parent patterns with the basic building patterns. This method first finds the instances of basic building patterns. It then iteratively increases the size of the patterns by joining the instances of the current set of patterns with the instances of basic building patterns. In each step of the join operation, non‐overlapping instances of network motifs are obtained by solving the maximum independent set problem, which is known to be NP‐complete. This cost is reduced by algebraically computing the overlapping instances. Algorithm 8 (see Fig. 18) presents the pseudocode of this method. This method can successfully discover network motifs up to size 15 using the exact census. This method is accurate and faster than Substructure Discovery (SUBDUE) and Frequent SubGraph mining (FSG).

*Limitation*: This algorithm is applied only for undirected networks. The runtime of this algorithm is not compared with state‐of‐art algorithms.

*ParaMODA*: ParaMODA improves upon the motif‐centric tool Grochow–Kellis and MODA [60]. The discovered motif instances can be saved in the disk for future analysis. This method can successfully discover network motifs up to size 7 using the exact census. ParaMODA is much faster than benchmark algorithms for specific query graphs. For all test cases, ParaMODA performs better than Grochow–Kellis and MODA.

*Limitation*: This algorithm is applied only for undirected networks. The speedup achieved in runtime depends upon the structure of the query graph.

*NemoMap*: Grochow–Kellis and MODA are extended to implement NemoMap algorithm [61]. This method can successfully discover network motifs up to size 7 using the exact census. NemoMap is faster than its predecessor for complex networks due to the better node selection process in symmetry‐breaking technique. NemoMap outperforms Grochow–Kellis and ParaMODA in all cases except simple patterns.

*Limitation*: This algorithm is applied only for undirected networks. Grochow–Kellis is better than NemoMap for simple query graphs with high symmetry.

*MODET*: MODET is based on a pattern growth approach that uses an ET [62]. Each node of ET represents a size‐*k* query graph. The frequency of a particular query graph is computed in a bottom‐up approach starting from the root node. The root node of ET represents a size‐3 tree whose frequency can be calculated explicitly from the input network. For motif size *k*, the ET first extended by adding vertices to the parent node to reach a size‐*k* tree, and then the edges are added to achieve a complete graph of size‐*k*. The embeddings of the corresponding nodes are computed from the embeddings of their parent node using a tree census and graph census procedure, respectively. This algorithm outperforms most of the benchmark algorithms.

*Limitation*: This algorithm uses a static ET that needs unbearable storage when motif size exceeds 10.

*MDET*: MDET uses a DET in place of a static ET and overcomes the space limitation of the MODET [63]. The growth of DET is controlled by pruning criteria applicable to the F2 measure. MDET can discover large network motifs up to size‐15 faster than benchmark algorithms.

*Limitation:* Although the DET required less storage than a static tree, the memory requirement cannot be satisfied when the motif size exceeds 15.

*Pattern‐join*: This algorithm uses a set of basic building patterns and applies pattern‐join operations iteratively to discover non‐overlapping motif instances of large patterns [64]. The exponential growth of the number of patterns with the increase of motif size is controlled by the downward closure property of F2 and F3 measures. This method outperforms the benchmark algorithms and able to discover network motifs up to size‐15 on the transcription network and PPI network.

*Limitation*: The pattern‐join algorithm can only find edge‐disjoint embeddings, and this algorithm is not applicable for F1 frequency measure. It consumes a lot of space for storing the embeddings of the parent pattern for large motifs, which restricts its usage for further increase in motif size.

## 7 Dataset and result analysis

There is a wide range of network datasets available for evaluating the network motif discovery tools and algorithms. The biological network plays a vital role in this field. Other relevant datasets include social networks, electronic circuits, food web, dictionary, power grid network, computer network, and WWW network. A single dataset may be used by some algorithms, whereas some other algorithms may use a wide variety of datasets. However, almost every tool and algorithms use at least one biological network dataset.

**Fig. 19:**
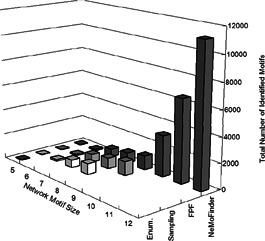
*Number of network motifs found in the PPI network of S. cerevisiae varying motif size (courtesy of Chen et al.* [54 *])*

**Fig. 20:**
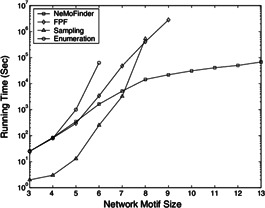
*Comparison of computational times to find network motifs of varying sizes in a PPI network of S. cerevisiae (courtesy of Chen et al.* [54 *])*

**Fig. 21:**
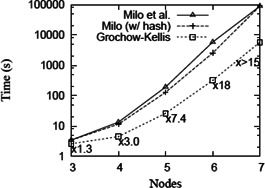
*Comparison of runtimes for different network motif sizes for the Grochow–Kellis algorithm and two versions of Milo et al. algorithm* [1 *] in the PPI network of S. cerevisiae (courtesy of Grochow and Kellis* [16 *])*

**Fig. 22:**
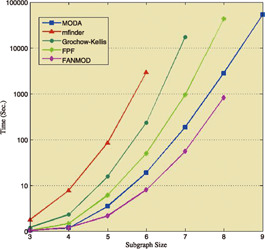
*Comparison of the runtime of MODA, MFinder, Grochow–Kellis, FPF, and FANMOD by varying subgraph sizes 3–9 in an E. coli transcription network (courtesy of Omidi et al.* [42 *])*

**Fig. 23:**
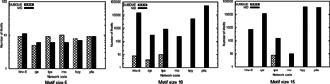
*Number of unique motif topologies found in PPI networks of six different species for motif sizes 5, 10, and 15 by Motif Discovery (MD) (Elhesha and Kahveci) and SUBDUE (courtesy of Elhesha and Kahveci* [59 *])*

**Fig. 24:**
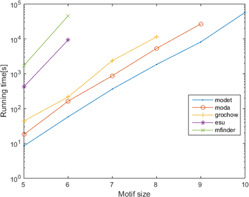
*Runtime of MODET compared with MFinder, ESU, Grochow–Kellis, and MODA for the PPI network of S. cerevisiae (courtesy of Patra and Mohapatra* [62 *])*

**Fig. 25:**
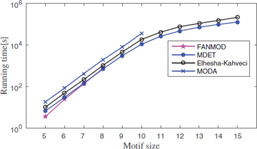
*Runtime of MDET compared with MODA, Elhesha–Kahveci, and FANMOD on the PPI network of S. cerevisiae (courtesy of Patra and Mohapatra* [63 *])*

**Fig. 26:**
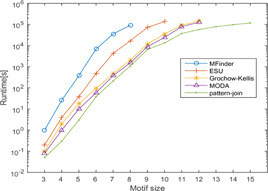
*Runtime of pattern‐join compared with MFinder, ESU, Grochow–Kellis, and MODA on a real network of S. cerevisiae (courtesy of Patra and Mohapatra* [64 *])*

Results taken from various tools and algorithms are presented here. Data is analysed based on the number of network motifs detected and the capacity of algorithms to discover the most abundant motif in reasonable running time. Runtime and memory comparison done by various tools and algorithms are analysed. Some experimental results taken from the literature can be seen in Figs. 19, 20, 21, 22, 23, 24, 25–26 and Tables 4, 5, 6, 7, 8, 9, 10, 11, 12–13. Owing to differences in numbers of generated random networks, computational environment, and different datasets, each figure and table should be examined separately.

**Table 4:** Network motifs in biological and technological networks; each table entry contains the number of subgraphs (*z*‐score). *z*‐score is determined by comparing with 1000 randomised networks (Courtesy of Milo *et al.* [1])

Network	Feed‐forward loop	Bi‐fan	Bi‐parallel	Three chain
*E. coli*	40(10)	203(13)	—	—
*S. cerevisiae*	70 (14)	1812 (41)	—	—
*C. elegans*	125 (3.7)	127 (5.3)	227 (20)	—
food of web	—	—	1357 (23)	1182 (7.2)
electronic circuit	424 (285)	1040 (1200)	480 (335)	—

**Table 5:** Number of subgraphs and respective subgraph classes detected by FANMOD by varying subgraph sizes from 3 to 6 (Courtesy of Wernicke [53])

Network	Subgraphs				Subgraphs’ classes			
	3	4	5	6	3	4	5	6
*E. coli*	5206	83,893	1,433,502	22,532,584	4	17	83	390
*S. cerevisiae*	13,150	183,174	2,508,149	32,883,898	7	33	173	888
*C. elegans*	47,332	1,394,259	43,256,069	1,309,307,357	13	197	7071	286,375
food of web	9487	169,733	2,908,118	45,889,039	8	57	629	9339

**Table 6:** Total number of subgraphs of different sizes in different networks detected by Kavosh (courtesy of Kashani *et al.* [41])

Network	3	4	5	6	8	10
*E. coli*	2590	12,896	80,724	558,080	29,294,103	1,529,707,241
*S. cerevisiae*	13,150	183,174	2,508,149	32,883,898	5,184,710,063	700,928,564,818
social	488	2183	10,599	52,156	1,224,376	26,429,201
electronics	1121	4316	19,675	97,038	2,572,125	71,614,362

**Table 7:** Number of non‐isomorphic subgraphs of different sizes in different networks detected by Kavosh (courtesy of Kashani *et al.* [41])

Network	3	4	5	6	7	8	9	10
*E. coli*	12	83	590	3884	23,587	136,569	768,121	4,223,040
*S. cerevisiae*	7	34	174	888	4809	27,003	183,307	1,083,282
social	13	108	773	5062	30,217	165,958	854,023	4,161,577
electronics	4	13	49	199	907	433	20,692	96,483

**Table 8:** Performance comparisons between Kavosh, FANMOD, MAVisto, and MFinder using the *E. coli* network, times are in seconds (courtesy of Kashani *et al.* [41])

Method	3	4	5	6	7	8	9	10
Kavosh	0.30	1.84	14.91	141.98	1374	13,174	121,110	1,120,560
FANMOD	0.81	2.53	15.71	132.24	1206	9256	—	—
MAVisto	13,532	—	—	—	—	—	—	—
MFinder	31	297	23,671.8	—	—	—	—	—

**Table 9:** Performance comparisons between Kavosh, FANMOD, MAVisto, and MFinder using the *S. cerevisiae* network, times are in seconds (courtesy of Kashani *et al.* [41])

Method	3	4	5	6	7	8	9	10
Kavosh	1.35	34.59	1004	20,213	746,386	17,111,178	3 × 10^8^	7 × 10^9^
FANMOD	2.20	41.41	1111	24,292	926,745	18,851,135	—	—
MAVisto	15,784	—	—	—	—	—	—	—
MFinder	32	306	33,548	—	—	—	—	—

**Table 10:** Runtime comparison of G‐trie with FANMOD and Grochow–Kellis on the social network, PPI network and electronic circuits. Runtime is measured in seconds (courtesy of Ribeiro *et al.* [55])

Network	Motif size	No. of subgraphs	Census on original network			Average census on random networks		
			FANMOD	Grochow–Kellis	G‐trie	FANMOD	Grochow–Kellis	G‐trie
social	3	2	0.31	0.11	0.02	0.35	0.11	0.02
	4	6	7.78	1.37	0.56	13.27	1.86	0.57
	5	21	208.30	31.85	14.88	531.65	62.66	22.11
yeast	3	2	0.47	0.33	0.02	0.57	0.35	0.02
	4	6	10.07	2.04	0.36	12.90	2.25	0.41
	5	21	268.51	34.10	12.73	400.13	47.16	14.98
circuit	6	33	0.49	0.41	0.03	0.55	0.24	0.03
	7	89	3.28	3.73	0.22	3.53	1.34	0.17
	8	293	17.78	48.00	1.52	21.42	7.91	1.06

**Table 11:** Memory limitation of QuateXelero (courtesy of Khakabimamaghani *et al.* [58])

Network	Motif size	Algorithm	Stopping reason
*S. cerevisiae*	9	G‐tries	long run time (close to 11 days)
	10	QuateXelero	long run time (about 26 days)
social	12	G‐tries	memory
	12	QuateXelero	memory
*E. coli*	12	G‐tries	memory
	11	QuateXelero	memory
electronic	12	G‐tries	core dumped
	13	QuateXelero	memory

**Table 12:** Number of significant motifs for six different PPI networks (courtesy of Patra and Mohapatra [62])

Motif size	Hhv8	Hhv1	*E. coli*	*H. pylori*	*R. norvegicus*	*S. cerevisiae*
5	16	20	7	15	15	21
6	59	70	16	62	69	110
7	181	212	38	354	381	612
8	1450	1508	91	2649	2450	3487
9	7101	12,351	235	15,382	8101	19,151
10	31,836	50,274	565	58,905	41,836	90,240

**Table 13:** Number of significant motifs for six different PPI networks (courtesy of Patra and Mohapatra [63])

Motif size	Hhv8	Hhv1	*E. coli*	*H. pylori*	*R. norvegicus*	*S. cerevisiae*
5	10	9	11	9	8	10
10	5368	4219	5718	3241	2816	4065
15	8152	7529	8418	6719	5245	7916

MFinder was evaluated on six different networks: the transcription network of *E. coli* (424 nodes and 519 edges) and *Saccharomyces cerevisiae* (685 nodes, 1052 vertices), the neural network of *Caenorhabditis elegans* (252 nodes, 509 edges), electronic circuits (10,383 nodes, 14,240 edges), WWW network (325,729 nodes, 1,460,000 edges), and food web of birds, fishes, and invertebrates (83 nodes, 391 edges) [1]. Network motifs found in various networks are shown in Table 4. The sampling method of MFinder is significantly faster than the exhaustive enumeration method. It is able to estimate the subgraph concentration at very high accuracy even for subgraphs that have low concentration. The MFinder can detect motifs up to size 7 [1].

The FPF algorithm in MAVisto [44] was tested only on a transcription network of *S. cerevisiae* (62 nodes, 93 edges). The performance of MAVisto was not compared with other tools and algorithms. MAVisto can detect network motifs up to size 7.

The RAND‐ESU algorithm in FANMOD [53] was evaluated on four different networks: the transcription network of *E. coli* (423 nodes, 519 edges), the transcription network of *S. cerevisiae* yeast (688 nodes, 1079 vertices), the neural network of *C. elegans* (306 nodes, 2345 edges), and the food web of the Ythan estuary (135 nodes, 597 edges). The number of subgraphs and the number of respective subgraph classes that occur in various networks are shown in Table 5. RAND‐ESU is much faster than edge sampling algorithm for subgraph sizes ≥5.

NeMoFinder was evaluated on two real‐time datasets of *S. cerevisiae* taken from the Uetz dataset and MIPS CYGD dataset [54]. The number of network motifs that can be found by NeMoFinder and other competitive algorithms is shown in Fig. 19. Run‐time comparison of NeMoFinder with FPF and MFinder is shown in Fig. 20. NeMoFinder can detect motifs up to size 13 with 20–100‐fold speed up as compared to the predecessor.

Grochow–Kellis algorithm was evaluated on the PPI network of *S. cerevisiae* (1379 nodes, 2493 edges) and the transcription network of *S. cerevisiae* (685 nodes, 1052 edges) [16]. This algorithm introduces the mapping of query graphs with symmetry‐breaking conditions. The number of subgraphs encountered without symmetry‐breaking is up to 100 times more than with symmetry‐breaking. The runtime comparison of the Grochow–Kellis algorithm with two versions of Milo *et al.* algorithm is shown in Fig. 21. Grochow–Kellis achieve an exponential improvement in runtime over the two versions of Milo *et al.* algorithm.

Kavosh was evaluated on four different networks: the metabolic pathway of *E. coli* (672 nodes, 1276 edges), the transcription network of *S. cereviciae* (688 nodes, 1079 edges), the real social network (67 nodes, 182 edges) and the electronic network (97 nodes, 189 edges) [41]. Kavosh performance was compared with MFinder, MAVisto, and FANMOD. The total number of subgraphs and the number of non‐isomorphic subgraphs of different sizes in different networks detected by Kavosh are shown in Tables 6 and 7, respectively. The runtime comparison of Kavosh with FANMOD, MAVisto, and MFinder for *E. coli* and *S. cerevisiae* network is shown in Tables 8 and 9, respectively. Performance of Kavosh is comparable to FANMOD, but it outperforms other tools.

MODA was tested only on the transcription network of *E. coli* (423 nodes and 519 edges) [42]. MODA was assessed for its computational time for enumerating subgraph appearances. Its runtime was compared with Grochow–Kellis, MFinder, FANMOD, and MAVisto for subgraphs of sizes 3–9 as shown in Fig. 22. MODA can discover network motifs by both exact census and sampling of subgraphs.

G‐trie was evaluated on a variety of networks: the dolphins social network (62 nodes, 159 edges), the electronic circuit (252 nodes, 399 edges), the social network (1000 nodes, 7770 edges), the PPI network of yeast (2361 nodes, 6646 edges), and the power grid network (4941 nodes, 6594 edges) [55]. Runtime comparison of g‐trie with FANMOD and Grochow–Kellis for electronic circuits, social network, and PPI network of yeast is shown in Table 10. G‐trie outperforms FANMOD and Grochow–Kellis for all networks. G‐trie can detect motifs up to size 9 in efficient running times.

NetMODE was tested on four different networks: the social network (67 nodes, 182 vertices), the metabolic pathway of *E. coli* (672 nodes, 1276 vertices), the transcription network of S. cerevisiae yeast (688 nodes, 1079 edges), and the complete directed graph (50 vertices, 2540 vertices) [56]. The runtime of NetMODE is compared with Kavosh and FANMOD on social network (4‐node) and transcription network of *S. cerevisiae* yeast (6‐node) under various switching methods [56]. NetMODE achieves better runtime performance for both the yeast and social networks.

Acc‐Motif was evaluated on various networks selected from Uri Alon's datasets and Pajek datasets [57]. The runtime of Acc‐Motif is compared with FANMOD to count isomorphic patterns of sizes 3 and 4 in a processed graph. Acc‐Motif achieves significant speedup over FANMOD for motif sizes 3 and 4.

QuateXelero was evaluated on six networks of different types: the transcription network of *S. cerevisiae* (688 nodes, 1079 edges), the metabolic pathway of *E. coli* (672 nodes, 1275 edges), the PPI network of the budding yeast (2361 nodes, 6646 edges), the real social network (67 nodes, 182 edges), the dolphins’ social network (62 nodes, 159 edges) and the electronic circuit (252 nodes, 399 edges) [58]. The performance of QuateXelero is compared with Kavosh and g‐tries on various networks above with different motif size ranges for the target network and random networks. The processing time of QuateXelero is better than Kavosh. However, it uses a massive amount of memory compared with Kavosh. QuateXelero is much faster than g‐tries for a census on the original network, but the census in random networks is slow for large motifs. Memory limitation and impractical running time of QuateXelero and g‐tries on various networks are shown in Table 11.

Elhesha–Kahveci algorithm was evaluated on real PPI networks of seven organisms taken from the MINT database: human herpes virus 8 (Hhv8; 48 nodes, 82 edges), *Campylobacter jejuni* (109 nodes 117 edges), *Treponema pallidum* (108 nodes 173 edges), *Rattus norvegicus* (535 nodes, 643 edges), *Helicobacter pylori* (717 nodes, 1472 edges), *E. coli* (616 nodes, 1561 edges), and *Plasmodium falciparum* (1221 nodes, 2577 edges) [59]. The number of unique motif topologies found by Elhesha–Kahveci in PPI networks of different species for motif sizes 5, 10, and 15 is compared with SUBDUE [78], as shown in Fig. 23. Runtime of Elhesha–Kahveci is compared with FSG [66] by varying motif sizes from 7 to 9 for various PPI networks. Elhesha–Kahveci was able to detect large network motifs with high frequency on dense input networks while SUBDUE was unable to achieve that result. For large motifs, Elhesha–Kahveci is much faster than FSG.

The runtime of ParaMODA is compared with Grochow–Kellis and MODA on the PPI network of *E. coli* and *S. cerevisiae* by varying motif sizes from 3 to 7 [60]. For all test cases, ParaMODA performs better than Grochow–Kellis and MODA. The runtime of NemoMap is compared with Grochow–Kellis and ParaMODA on the PPI network of *Homo sapiens*, *E. coli*, and *S. cerevisiae* by varying motif sizes from 4 to 7 [61]. NemoMap outperforms Grochow–Kellis and ParaMODA in all cases except simple patterns.

MODET was evaluated on real PPI networks of six organisms taken from the MINT database: Hhv8 (92 nodes, 170 edges), Hhv1 (176 nodes, 353 edges), *E. coli* (402 nodes, 727 edges), *H. pylori* (738 nodes, 1643 edges), *R. norvegicus* (1825 nodes, 3471 edges), and *S. cerevisiae* (3187 nodes, 9171 edges) [62]. The number of network motifs found in the above networks for motif sizes 5–10 is shown in Table 12. The runtime of MODET is compared with MFinder, ESU, Grochow–Kellis, and MODA for the PPI network of Hhv8, *E. coli*, and *S. cerevisiae*. The result of the *S. cerevisiae* network is shown in Fig. 24. MODET is significantly faster than state‐of‐art algorithms.

MDET was also evaluated on real PPI networks of six organisms taken from the MINT database [63]. The number of network motifs found in the above networks for motif sizes 5, 10, and 15 is shown in Table 13. The runtime of MDET is compared with FANMOD, Elhesha–Kahveci, and MODA for the PPI network of Hhv8, *E. coli*, and *S. cerevisiae*. The result of the *S. cerevisiae* network is shown in Fig. 25 MDET is significantly faster than most of the existing motif finding algorithms, and the use of DET eliminates the memory limitation of the static ET.

The pattern‐join algorithm is tested on the transcription regulatory network of *E. coli* (116 Transcription Factors (TFs) and 423 operons, 578 interactions), PPI network of *S. cerevisiae* (858 proteins, 1815 interactions) and Hhv8 (92 proteins, 170 interactions) [64]. The runtime of pattern‐join is compared with MFinder, ESU, Grochow–Kellis, and MODA for the above networks. The result obtained from the *E. coli* network is shown in Fig. 26. Pattern‐join outperforms most of the existing algorithms for all the above networks.

Some of the parameters to analyse the above tolls and algorithms are discussed below.

*Accuracy*: All exact census algorithms detect 100% of the available network motifs up to their size limit through exhaustive recursive search. However, the sampling algorithms are able to identify relatively large motifs by compromising accuracy. Exact census algorithms are MAVisto, NeMoFinder, Grochow–Kellis, Kavosh, g‐tries, NetMODE, Acc‐Motif, QuateXelero, Elhesha–Kahveci, ParaMODA, NemoMap, MODET, MDET, and Pattern‐join etc. Accuracy of the algorithms that follow sampling approaches such as MFinder, FANMOD, and MODA is <100%. MFinder produces biased results, whereas FANMOD is less biased than MFinder. The sampling strategy of MODA is also biased, and hence accuracy cannot meet 100%.

*Runtime efficiency*: The efficiency of network motif discovery algorithms can be measured in terms of runtime. Usually, runtime depends on the size of the network motif and the size of the input network. However, the strategy of the algorithms plays a vital role in deciding runtime. For example, the sampling strategy used by MFinder and FANMOD is faster than their respective exhaustive enumeration. Runtime efficiency of the existing algorithms can be analysed from their relative performance. For example, NeMoFinder achieves 20–100‐fold speed up over FPF and MFinder. Grochow–Kellis attain an exponential improvement in runtime over MFinder. Runtime efficiency of Kavosh is comparable to FANMOD, but it outperforms MFinder and MAVisto. Runtime efficiency of MODA is better than Grochow–Kellis, MFinder, and MAVisto. G‐tries outperforms FANMOD and Grochow–Kellis over runtime. NetMODE shows better runtime performance than Kavosh and FANMOD. Acc‐Motif attains a significant speedup over FANMOD. QuateXelero is better than Kavosh and g‐tries in terms of runtime. Elhesha–Kahveci is much faster than FSG. ParaMODA performs better than Grochow–Kellis and MODA. NemoMap outperforms Grochow–Kellis and ParaMODA. MODET is faster than MFinder, ESU, Grochow–Kellis, and MODA. MDET is significantly faster than MODA and Elhesha–Kahveci. Pattern‐join outperforms MFinder, ESU, Grochow–Kellis, and MODA.

*Computing store*: The computing store increases exponentially with respect to motif size and network size. The memory requirement also depends on the strategy used by the tools and algorithm. MFinder required a large space to maintain the associated hash tables. Algorithms that run out of space before running out of time are MODA, MODET, Elhesha–Kahveci etc. The memory requirement for the data structure used to store the subgraphs in MODA, g‐tries, QuateXelero, and MODET becomes impractical for motif size beyond their limit. A relative comparison among the algorithms is helpful for the analysis. For example, Kavosh mentioned that its memory usage is considerably less than FANMOD. Enormous memory requirements of NetMODE to store parent patterns slow down the kernel. ParaMODA frequently runs out of memory and crashes. MODET runs out of space to save more than a size‐10 ET. MDET overcomes the space limitation of MODET significantly.

*Scalability*: The scalability of a motif discovery algorithm is measured with respect to motif detection capability and the size of the input network. Some algorithms are scalable with respect to both network‐size and motif‐size, such as NeMoFinder, Kavosh, Elhesha–Kahveci, MDET and pattern‐join. Grochow–Kellis and QuateXelero have limited scalability. MODA, g‐tries, and MODET are scalable only with respect to network size. MFinder and FANMOD have limited scalability only with respect to network size. Some algorithms are not at all scalable such as NetMODE, Acc‐Motif, ParaMODA, and NemoMap.

Table 14 presents the motif detection capability of the above tools and algorithms.

**Table 14:** Motif detection capability of the network motif discovery tools and algorithms

Tools/algorithms	Size of the largest motif found	Census type	Network type
MFinder [1]	8	sampling	directed
MAVisto [44]	7	exact‐census	directed
FANMOD [53]	8	sampling	directed
NeMoFinder [54]	13	exact‐census	undirected
Grochow and Kellis [16]	7	exact‐census	directed
Kavosh [41]	12	exact‐census	directed
MODA [42]	10	sampling	undirected
G‐tries [55]	9	exact‐census	undirected
NetMODE [56]	6	exact‐census	directed
Acc‐Motif [57]	5	exact‐census	directed
QuateXelero [58]	12	exact‐census	directed
Elhesha–Kahveci [59]	15	exact‐census	undirected
ParaMODA [60]	7	exact‐census	undirected
NemoMap [61]	7	exact‐census	undirected
MODET [62]	10	exact‐census	undirected
MDET [63]	15	exact‐census	undirected
pattern‐join [64]	15	exact‐census	directed

Each of the above algorithms has merits and demerits with respect to specific criteria. For example, Acc‐Motif is the best tool for small motifs size in the range of 3–5. FANMOD is an excellent tool for motif size up to 8 because of its runtime efficiency. However, it does not apply to large motifs. MODA and MODET are superior for undirected graphs up to size 10. For directed graphs, QuateXelero gives the best runtime efficiency up to motif size 12. Elhesha–Kahveci or MDET can be used for undirected graphs and non‐overlapping motif instances up to size 15.

## 8 Conclusions and future work

Advancement in science and technology produces a massive volume of real networks in various fields. The analysis of these networks gives an insight into the corresponding systems and organisms. The overrepresented subgraphs in these networks, which are statistically significant, are called network motifs. Network motifs are the building blocks of networks and are often biologically significant, which makes the identification of the motifs extremely important. Network motif discovery has proved to be a computationally challenging task. There exist many tools and algorithms to discover network motifs. However, motif discovery capabilities can be improved further with new developments.

The network motif discovery problem mainly includes subgraph isomorphism check, random graphs generation, subgraph frequency counting in the networks by sampling strategy, or exact census. Each of these factors has challenges and possibilities that can be listed below:

- The exponential increase of computational resources with respect to both graph and motif size for enumerating subgraphs prohibits tools and algorithms from dealing with large motifs.
- Solving an NP‐complete problem for the subgraph isomorphism check is highly expensive.
- Determine the statistical significance of a candidate motif required repeated computations in a sufficient number of randomised networks.
- The challenges mentioned above increase further due to the continuous growth of real‐world networks.

In this study, the state‐of‐art strategies for discovering network motifs are discussed with their strengths and limitations. The algorithms are explained with pseudocode and classified based on their principles or approaches. Algorithms are primarily classified as sampling algorithms and exact census algorithms. The sampling strategies are used to accelerate the enumeration process by compromising accuracy. Motif‐centric methods such as Grochow–Kellis and MODA are faster than others for small query graphs. However, network‐centric approaches are preferable as the size of the motif increases. The major algorithms in the literature are MFinder, MAVisto, FANMOD, Nemofinder, Grochow–Kellis, Kavosh, MODA, QuateXelero, Elhesha–Kahveci, MODET, and MDET. Results of various experimental runs carried out using different network motif discovery tools have helped to determine which tools are more efficient and useful. Furthermore, these comparisons help to highlight which algorithmic methods improve tool performance.

Most of the algorithms that exist in the literature can find motifs in the single‐digit range. Hence efficient and scalable algorithms are required to discover large motifs. There is a continuous improvement in network motif discovery tools and algorithms throughout the years. However, there is a scope for further improvements in this field. The following research topics can be studied in detail for the future development of network motif discovery.

- Small motifs are the constituents of large motifs; hence the small motifs can be used as a seed to search the large motifs. This idea can be used to design scalable algorithms for discovering large motifs.
- The number of redundant computations in the random networks can be reduced significantly by limiting the subgraph census only to a small set of potential motifs. Efficient data structures need to be developed to track the candidate motifs.
- Colour coding techniques can be applied to quickly find network motifs in the input network as well as random networks.
- Hybrid algorithms can be proposed by combining the efficient parts of two or more algorithms.
- Polynomial‐time algorithms do exist for subgraph isomorphism check for special patterns. These distinctive patterns can be prioritised in the motif discovery process.
- Parallel algorithms can be implemented to decrease the runtime. Some existing algorithms have exploited only coarse grain parallelism. Efficient fine‐grained parallelism is perhaps the most crucial improvement needed currently for network motif discovery that could simultaneously analyse different parts of the network, thus reducing the execution time and enabling our reach to larger motifs and networks.
- The future version of the tools most includes a user‐friendly interface for better visualisation and analysis.
- The tools and algorithms most support a wide variety of input/output formats.
- Web tools are more convenient to use in comparison with installing the tool locally because the tools may require some resources which are not available in the local machine.
